# Supplementary material for: From Bench to Bedside: Clinical and Biomedical Investigations on Hepatitis C Virus (HCV) Genotypes and Risk Factors for Albuminuria
Source: Bioengineering (Basel). 2022 Sep 27;9(10):509. doi: 10.3390/bioengineering9100509 (PMC9598589; doi:10.3390/bioengineering9100509)
Supplement: Supplementary file 1 [file bioengineering-09-00509-s001.zip › bioengineering-1859357-supplementary.pdf]

Supplementary Table S1. Liver function tests and lipid profiles in the distribution of HCV genotypes

	HCV-RNA Positive	HCV-RNA Negative	*p* -value	Genotype 1a	Genotype 1b	Genotype 2	Genotype 3	*p* -value
AST (U/L)	61.49 (2.782)	27.41 (1.508)	<0.001***	60.18 (2.974)	67.07 (10.192)	48.30 (5.462)	70.41 (8.509)	0.601
ALT (U/L)	64.54 (4.567)	24.60 (1.499)	<0.001***	60.41 (2.971)	76.23 (21.886)	54.60 (8.390)	78.41 (11.404)	0.811
Total bilirubin (mg/dL)	0.7312 (0.02)	0.6454 (0.025)	0.991	0.7172 (0.0267)	0.7459 (0.03881)	0.7100 (0.05700)	0.7852 (0.0680)	0.797
LDL (mg/dL)	94.75 (5.461)	114 (9.809)	0.320	90.95 (7.368)	101.67 (13.745)	101.67 (6.766)	93.67 (1.764)	0.857
HDL (mg/dL)	56.03 (2.319)	49.66 (3.186)	0.386	56.85 (3.407)	55.29 (3.395)	50.29 (6.301)	50.40 (3.027)	0.317
Cholesterol (mg/dL)	175.9 (2.144)	187.47 (2.848)	0.255	175.39 (2.745)	178.48 (5.249)	192.00 (6.805)	160.33 (6.518)	0.086
Triglyceride (mg/dL)	135.36 (5.331)	157.87 (10.6)	0.039	138.55 (7.115)	146.39 (13.729)	134.53 (15.281)	106.37 (11.824)	0.544

*** *p*-value <0.001.

Abbreviations: AST: Aspartate Aminotransferase; ALT: Alanine Aminotransferase; HDL: high-density lipoprotein; LDL: low-density lipoprotein.
